# Next-Generation Cancer Biomarkers: Extracellular Vesicle DNA as a Circulating Surrogate of Tumor DNA

**DOI:** 10.3389/fcell.2020.622048

**Published:** 2021-02-02

**Authors:** Samuel Amintas, Véronique Vendrely, Charles Dupin, Louis Buscail, Christophe Laurent, Barbara Bournet, Jean-Philippe Merlio, Aurélie Bedel, François Moreau-Gaudry, Julian Boutin, Sandrine Dabernat, Etienne Buscail

**Affiliations:** ^1^Département des Sciences Biologiques et Médicales, Université de Bordeaux, Bordeaux, France; ^2^U1035 Institut National de la Santé et de la Recherche Médicale (INSERM), Bordeaux, France; ^3^Centre Hospitalier Universitaire (CHU) de Bordeaux, Bordeaux, France; ^4^Centre Hospitalier Universitaire (CHU) de Toulouse, Toulouse, France; ^5^INSERM UMR 1037, Toulouse Centre for Cancer Research, University of Toulouse III, Toulouse, France; ^6^INSERM U1053, Bordeaux, France; ^7^INSERM, UMR-1220, IRSD, University of Toulouse III, Toulouse, France

**Keywords:** circulating biomarker, cancer, liquid biopsy, extracellular vesicles, microvesicles, exosomes, EV-DNA

## Abstract

Extracellular vesicles (EVs) are produced by healthy tissues and tumor cells and are released in various bodily fluids, including blood. They are limited by bilayer phospholipidic membranes, and they carry a rich content in biomolecules. Their release cleanses the cells of their waste or serves as functional local and distant cell–cell communication and molecular exchange particles. This rich and heterogeneous content has been given intense attention in cancer physiopathology because EVs support cancer control and progression. Because of their specific active cargo, they are being evaluated as carriers of liquid biopsy biomarkers. Compared to soluble circulating biomarkers, their complexity might provide rich information on tumor and metastases status. Thanks to the acquired genomic changes commonly observed in oncogenic processes, double-stranded DNA (dsDNA) in EVs might be the latest most promising biomarker of tumor presence and complexity. This review will focus on the recent knowledge on the DNA inclusion in vesicles, the technical aspects of EV-DNA detection and quantification, and the use of EV-DNA as a clinical biomarker.

## Introduction

Extracellular vesicles (EVs) have long been observed, but they were mostly considered as garbage bags to get rid of unusable intracellular constituents (Johnstone, 1992). In the mid-1990s, researchers started to report other functions for EVs. In B cells, internal vesicles rich in membrane major histocompatibility complex [MHC] class II could be released as small vesicles by exocytosis. They were considered to facilitate antigen presentation (Guescini et al., 2010). EVs could bring complex signals not only through their membrane but also by releasing the content of their lumen in the recipient cell, enabling horizontal transfer of proteins and RNAs (Cocucci et al., 2009). These concepts shed a new light on cell–cell communication, thought to occur until then only by soluble signals decoded upon receptor binding or by connexons. Within the EVs, exosomes were found vectors of intercellular communication in physiological and pathological conditions (reviewed by Kalluri, 2016). Consequently, a swift increase in EV literature occurred, and a lot of attention was given to the identification of the protein and RNA (including miRs) cargoed in EVs as biomarkers in cancer. Now, another important type of EV biomolecules emerges as relevant for cancer biology: the EV-DNA. The aim of this review is not to propose a complete description of EV generation, release, and outcome but to focus on their DNA content as a valuable material for cell–cell communication and a relevant biomarker in cancer clinical biology.

## Circulating Cell Free DNA and EVs

**FIGURE 1:**
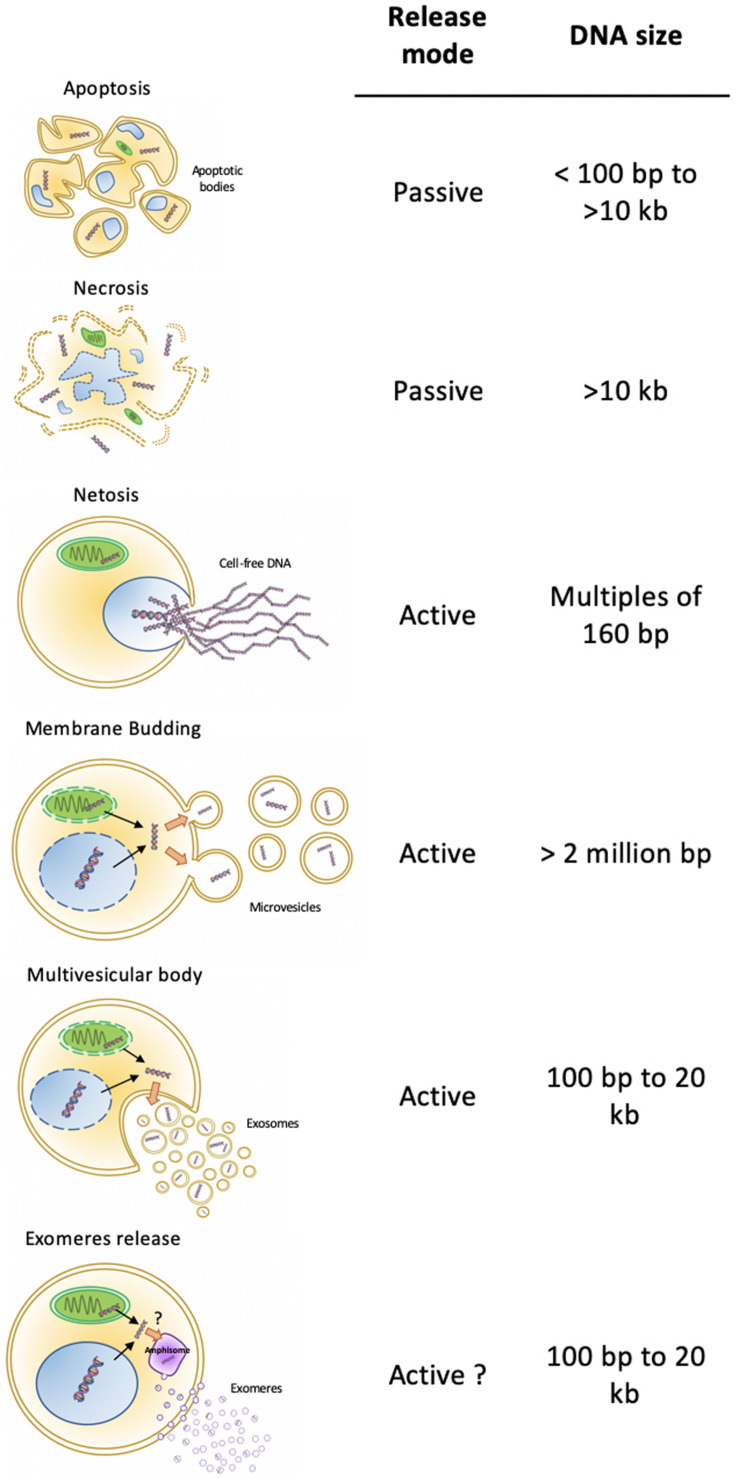
Cell-free DNA cell sources. DNA can be released by active or passive mechanisms. Apoptosis is a passive mechanism releasing apoptotic bodies with fragmented genomic DNA, in which sizes encompass a range of 100 bp to more than 10 kb. Necrosis is a passive way of DNA release, with free and non-vesicular all sizes fragments. Active mechanisms of DNA release include NETosis, with projection of very long, decondensed genomic DNA, microvesicles budding from a plasma membrane, exosomes secretion from multivesicular bodies, and exomeres released from amphisomes. Depending on the active mechanism, DNA fragment length varies between 100 bp (exosomes and exomeres) and reaching more than 2 million bp (Microvesicles).

As liquid biopsies have been given more and more consideration for cancer patient management, a lot of attention and technical development aimed at detecting circulating cell free DNA (cfDNA), especially circulating tumor DNA (ctDNA) within total cfDNA. Circulating cfDNA, and a fortiori ctDNA, originates from healthy or tumor tissues. In plasma, it can be free, released from dying healthy or tumor cells (necrosis or necrosis-related programmed cell death; Figure 1). In addition, decondensed neutrophil DNA can be ejected by NETosis as a pathogen trap (Cahilog et al., 2020; Zhen et al., 2020). Otherwise, cfDNA can be embedded in bilayer lipid biomembranes. Besides the apoptotic vesicles, in which compaction favors dead cell disposal, two major modes of EV release coexist: the exocytosis of multivesicular bodies (MVBs) and shedding vesicles directly budded from the plasma membrane (Figure 1). These processes are considered active, and the content of the released vesicles may be selective. Vesicles from MVBs are also known as exosomes. These distinct origins diversify their content, including their DNA. In fact, plasma cfDNA concentration analysis showed that more than 93% of amplifiable cfDNA is located in plasma EVs (Fernando et al., 2017). Thus, EVs are potential valuable materials for circulating DNA-based biomarker discovery.

The size of circulating cfDNA differs according to its origin. Apoptotic bodies carry short genomic fragments of double-stranded DNA (dsDNA), in which sizes correspond to the DNA covered by nucleosomes, protected from the apoptosis-induced endonucleases (around 160–180 base pairs) (Bortner et al., 1995). Thus, any genomic locus, a byproduct of cell death, can be embedded in apoptotic vesicles under a highly compacted form (Serrano-Heras et al., 2019). Vesicle-free DNA ongoing non-specific degradation after necrosis-related cell death or NETosis presents wider ranges of lengths from less than 100 bp for mitochondrial DNA (mtDNA) to several thousands of bp (Figure 1). The fragments encompass the whole genome due to the non-specific destruction of the cell and its components. Interestingly, besides transferring various proteins, lipids, and RNAs to recipient cells, cultured tumor cells and tumor cells xenografted in mice also released EVs carrying DNA, which reflected the genetic status of the tumor, including the amplification of the oncogene c-Myc (Balaj et al., 2011). Exosomes can also work as vesicular carriers of mtDNA (Guescini et al., 2010). Exosome double-stranded DNA (exoDNA) from cell lines, with sizes ranging from 100 to 2.5 kbp, can represent the entire genome and reflect the mutational status of tumor parental cells (Thakur et al., 2014; Vagner et al., 2018). Moreover, EV-mediated horizontal high molecular weight DNA transfer might contribute to creating cellular diversity in healthy cells and promote cancer progression (Fischer et al., 2016). Although rare, the authors also observed a propagation of the transferred DNA to daughter cells, likely through an integrative event. These observations suggest that the DNA inclusion in EVs is a selective active process, dedicated to share specific parts of the tumor genome (Lázaro-Ibáñez et al., 2014). Interestingly, most of studies focused on dsDNA and made no mention of single-strand DNA (ssDNA) associated to EVs.

How DNA species are sorted into EVs is also far from being resolved. The inhibition of exosome secretion results in accumulation of nuclear DNA in the cytosol, provoking a senescence-like phenotype, with cell cycle arrest and eventually apoptosis (Takahashi et al., 2017). This suggests that DNA embedding in exosomes is important for keeping cell homeostasis. DNA secretion through exosomes protects tumor cells from the inflammatory reaction induced by a DNA-triggered stimulator of interferon response (STING) signaling (Harding et al., 2017; Le Naour et al., 2020). DNA fragments can arise from damaged genomic DNA, packaged in micronuclei, which are cytosolic vesicles enveloped by a nuclear membrane (Fenech et al., 2011). Following nuclear membrane collapse of micronuclei, released genomic DNA can interact with exosomal tetraspanins, leading to the shuttling of the damaged DNA in MVBs (Figure 1; Yokoi et al., 2019). The same study found that exoDNA reflected copy number variation of ovarian cancer primary tumors. In addition, vesicular mtDNA might arise from distinct vesicles produced by oxidized mitochondria reaching the endolysosomal system to form MVBs (reviewed in Picca et al., 2020). These EVs can, in turn, transfer their mtDNA to cells with impaired metabolism, leading to the restoration of metabolic activity and treatment resistance (Sansone et al., 2017).

Recent studies contradict the vesicular nature of cfDNA. Although very scarce, evidence shows that EV-DNA could be bound to the outer lipid layer of EV membranes (Németh et al., 2017). High-resolution density gradient fractionation, to avoid aggregation of vesicles and obtain a pure fraction of exosomes, showed that exosomes or any other type of small EVs actually did not contain any dsDNA nor histones H2A, H3, or H4 (Jeppesen et al., 2019). Instead, active secretion of DNA and histones occurred through an amphisome-dependent mechanism, involving CD63-positive multivesicular endosomes-like structures (MVE). Amphisomes are hybrid organelles appearing from the fusion of an autophagosome and an MVE. The authors propose a new model, yet to be confirmed, in which the dsDNA might be secreted by amphisome fusion to the plasma membrane in a non-vesicular way. This non-vesicular pathway could be the recently described nanoparticular exomeres actively secreted by cells and different from mere aggregates (Zhang et al., 2018). DsDNA was found in both exomere and exosome fractions. Whether exomere-DNA can be functional as the exomere protein cargo needs to be confirmed (Zhang et al., 2019). By contrast, fractions from high-resolution iodixanol density gradients were recently found positive for DNA fragments and exosome markers such as CD63, CD9, CD81, flotillin-1, and TSG101 (Lázaro-Ibáñez et al., 2019).

In conclusion, more work is needed to ascertain the embedding mechanism and the final location of DNA in EVs and nanoparticles.

## Technical Aspects

### Exosomes and EVs Isolation

To ascertain the presence of DNA in EV, the first step before analysis of EV-DNA is the correct and specific isolation of EVs. This is crucial to obtain pure material and to have minimal impact on DNA extraction and further analyses. Various isolation and purification methods for EVs have been recently reviewed in literature (Yang et al., 2020). Even if ultracentrifugation is still the gold standard for EV enrichment, alternative technologies allow for the purification of subpopulations of EVs such as exosomes. For instance, size exclusion chromatography seems to emerge as a promising method because it significantly limits vesicle structural damages, such as aggregation induced by high-speed centrifugation (An et al., 2018; Royo et al., 2020; Sidhom et al., 2020). This method separates vesicles according to their sizes, which is not necessarily related to their functions. Some authors report optimal vesicle enrichment and soluble protein removal with a combination of ultrafiltration and size exclusion chromatography (Diaz et al., 2018). Five different methods for isolation and separation of EVs from protein and lipid particles in human serum were compared. The authors concluded that sequential use of two or more techniques greatly improved the depletion of lipoprotein and protein contaminants but significantly reduced the yield of EVs (Brennan et al., 2020). The choice of the appropriate isolation method depends on the initial quantity of available material and the desirable amount of EVs.

Microfluidic platforms represent another auspicious technology in the field of EV research. In particular, in the line of single cell analysis, new integrated microfluidic techniques facilitate combinatorial exosome isolation and analysis based on their physical and biochemical properties (Yang et al., 2020). A recent publication describes a microfluidic device allowing the rapid isolation of tumor derived exosomes from the plasma of pancreatic ductal adenocarcinoma (PDAC) patients (Kamyabi et al., 2020). EVs or exosomes are captured by antibodies against CD63, CD9, and CD81 surface protein (exosome) or membrane EPCAM (tumor). The antibody/antigen bounds are then ruptured to recover the immobilized EVs. After DNA extraction, the authors were able to identify *KRAS* mutations by droplet digital PCR in EV-DNA. Of note, they recovered more total exoDNA in CD9/CD63/CD81 vesicles than in EPCAM-positive EVs, but this latter fraction contained more mutant *KRAS* DNA.

### EV-DNA Properties and Extraction Methods

Unlike cfDNA, EV-DNA is protected by a lipid bilayer membrane, which has a protective function against nucleases, thus increasing stability. Protected DNA is particularly adapted to lab routine analysis (Jin et al., 2016). Moreover, tumor EV-DNA amounts vary depending on distinct vesicle types separated by iodixanol density gradient. Like for protein and RNA, genomic DNA (gDNA) identified specific EV subpopulations also positive for exosomal proteins (Lázaro-Ibáñez et al., 2019). In the same way, different gDNA contents characterized apoptotic bodies, microvesicles, and exosomes (Lázaro-Ibáñez et al., 2014). After a differential centrifugation fractionation, the authors searched for specific genes and sequences within the different EV fractions. A 108 bp intron fragment for MLH1 was found in most EV types. Mutant *TP53* and *PTEN* suppressor gene sequences were present in apoptotic bodies, but in very small amounts in EVs and exosomes. Thus, it seems that higher DNA concentrations are associated with larger vesicles (Vagner et al., 2018; Lázaro-Ibáñez et al., 2019), and higher gDNA rates were packed in microvesicles from tumor as compared to normal cells (Balaj et al., 2011). Thus, paralleling the still obscure embedding mechanism of EV-DNA, identifying the best fractions for analysis remains challenging.

As mentioned in the previous chapter, gDNA was found associated to the surface of EVs, which may interfere with intravesicular DNA characterization. As extraneous DNA was found at the urinary exosome surface, a protocol to avoid contaminations and isolate internal gDNA only may be needed (Miranda et al., 2010). In the same way, DNase sensitive nucleic acids have been identified on the surface of mast cell EVs (Shelke et al., 2016) and EVs isolated from melanoma patients’ plasma (Zocco et al., 2020). Although intracytoplasmic uptake of those nucleic acids occurred in recipient cells, no observable functional impact was relayed intracellularly. More recently, Vagner et al. (2018) concluded that most of the DNA content was localized on the outside or the surface of EVs, with only a small portion being internalized in EVs, but again, the functionality of this surface DNA was not described.

Regardless of the analyzed vesicles, EV-DNA extraction slightly differs from conventional DNA extraction from cells or tissues but seems to be shared by many authors. Briefly, a lysis step followed by washing and elution on spin columns are often reported. EV-DNA extraction kits are now available from several commercial suppliers, with similar methods. Moreover, in order to avoid surface-associated DNA contamination, most procedures include a DNAse I or exonuclease I pretreatment.

In conclusion, all EV types seem to be distinct in their DNA content, but the sorting of the different loci and/or mutant DNA molecules is far from being understood. Additionally, heterogeneity resides in the definitions of EV fractions. Consensual agreement will define subcategories of large and small EVs, most likely by the presence of specific biomarkers, in order to better characterize their content and functional properties, as elegantly proposed recently (Jeppesen et al., 2019). On the technical aspect, future comparison between labs is needed to validate the full process of EV enrichment and DNA extraction.

## EV-DNA Reflects Tumor Genome Hallmarks

### Tumor Genome and Exome

After whole genome sequencing (WGS), human PDAC tumor genome DNA and exoDNA shared 92% of the reads, suggesting that exoDNA is representative of all chromosomes (Kahlert et al., 2014). Moreover, 65–91% coverage of the human genome was found in pancreaticobiliary cancer exosomes (San Lucas et al., 2016). In the same way, the murine tumor intestinal epithelial cell line RAS-3 EV-DNA covered more than 90% of the parental cell genome (Lee et al., 2014). Interestingly, exoDNA human sequences were 95–99% identical to the exomes of solid tumors (Vagner et al., 2018; Lázaro-Ibáñez et al., 2019). The same conclusions were drawn with serum exoDNA from pheochromocytoma (PCC) and paraganglioma (PGL) patients (Wang et al., 2018). Human prostate cancer exoDNA even spanned mtDNA (Vagner et al., 2018). Consistent with previous observations, WGS revealed that EV-DNA derived from human mast and erythroleukemic cell lines spanned all chromosomes and mtDNA (Lázaro-Ibáñez et al., 2019).

Taken together, these results confirm that exoDNA is at least representative of the entire tumor exome and may be even the whole genome.

### Tumor-Specific Sequence Variations

As carriers of tumor genome and/or exome, EV-DNA allows for the detection of specific gene mutations and gene amplification originally present in the primary tumor and its metastases. Based on preclinical results, multiple studies describe the identification of oncogenic alterations or tumor suppressor gene mutations in EV-DNA. For instance, Balaj et al. (2011) found typical *c-myc* amplification in EV-DNA extracted from cultured medulloblastoma cell lines as well as medulloblastoma tumor bearing mouse sera. Similarly, inactivating point mutations of tumor suppressors *TP53* and *PTEN* were identified in exoDNA from prostate cancer cell lines bearing these mutations (Lázaro-Ibáñez et al., 2014). Stepping toward clinical applications, Kahlert et al. (2014) identified matched *KRAS* and *TP53* hotspot mutations in serum exosomal DNA and solid tumor DNA from PDAC patients. The same observations were made for *RET*, *HIF2A*, *VHL*, and *SDHB* point mutations in PCCs and PGLs bared by patients or mice (Wang et al., 2018). More recently, androgen receptor gene (*AR*) amplifications have been identified in 12 of 15 patients from a castration-resistant prostate cancer (CRPC) cohort using DNA extracted from EV-enriched plasmas (Foroni et al., 2020; Table 1). The detection of *AR* T878A mutation was more challenging.

**TABLE 1:** Results of the main clinical studies assessing the performance of EV-DNA biomarkers.

**Mutation(s) detected**	**Stage of tumors: localized, metastatic, all stages**	**Mutant DNA localization**	**Type of sample**	**Clinical relevance and potential application**	**References**
**Ovarian cancer**					
*DROSHA*, *LIG4*, *MACROD2*, *SATB1*, *RASSF6*, and *BIRC2*	Metastatic	Internal double-stranded exoDNA	Ascites and plasma	Treatment with genotoxic drugs resulted in increased cancer cell micronuclei and genomic DNA and other nuclear contains into exosomes	Yokoi et al., 2019
**Glioblastoma**					
*IDH1G395A*	All stages (II–IV)	Internal and external	Peripheral blood and surgical tissue sample	*IDH1G395* mutation is detected in exosomes, correlation with diagnostic and prognostic in all stages, DNA-containing EVs can cross the disrupted blood–brain barrier	García-Romero et al., 2017
**Non-small cell lung cancer**					
*EGFR*	Advance non-small cell lung cancer	ExoDNA (internal) and ctDNA	Plasma and matched baseline plasma and tissue biopsy samples	Combining exoDNA and ctDNA increased the sensitivity for EGFR mutation detection in plasma. Useful in M0/M1a patients	Krug et al., 2018
*EGFR T790M*	All stages and healthy controls	ExoDNA/RNA (internal) and ctDNA	Plasma	The combination of exoDNA/RNA and ctDNA for *EGFR T790M* has a better sensitivity and specificity than ctDNA alone	Castellanos-Rizaldos et al., 2018
*EGFR*	All stages	ExoDNA (internal)	Plasma	Diagnostic and prognosis	Hur et al., 2019
*EGFR T790M*	All stages	ExoDNA (internal) and ctDNA	Plasma and bronchial washing	Diagnosis and prognosis	Park et al., 2020
**Urothelial carcinoma of bladder**					
*MDM2*, *ERBB2*, *CCND1*, *CCNE1*, *CDKN2A*, *PTEN*, *RB1*	T2, T3, T4, N0, N2	ExoDNA (internal) and ctDNA	Urine samples	Identification of somatic mutation and copy number variation using ctDNA and exoDNA in urine samples	Lee et al., 2018
**Prostate cancer**					
*P53*, *MLH1*, *PTEN*	T1c, T3	ExoDNA internal and external	Plasma	EVs contain dsgDNA fragments that could be used to detect specific mutation. EVs could be used as potential biomarkers for diagnostic and prognosis	Lázaro-Ibáñez et al., 2014
AR gene amplification, AR-V7 transcript, and T878A mutation	Metastatic castration-resistant prostate cancer	ExoDNA and exoRNA internal and external	Plasma	Selective isolation of a subset of circulating exosomes enriched for tumor origin increases sensitivity and specificity for the detection of specific alterations	Foroni et al., 2020
*MYC*, *PTEN*	Metastatic castration-resistant prostate cancer	Single-stranded and double-stranded DNA	Plasma	EVs contain extracellular DNA and suggest that it could be used to monitor metastatic prostate cancer	Vagner et al., 2018
**Melanoma**					
*BRAF ^*V600E*^*	T3, T4	ExoDNA (internal and external) and cfDNA	Plasma	Significant improvement in *BRAF ^*V600E*^* mutation detection combining cfDNA and EV-DNA analysis using peptide affinity assay	Zocco et al., 2020
**Pancreatic cancer**					
*KRAS*	All stages	ExoDNA (internal) and ctDNA	Plasma	Higher *KRAS* exo-DNA MAF was associated with decreased DFS in patients with localized disease	Allenson et al., 2017
*KRAS*^*G12D*^, *TP 53*^*R273H*^	Resectable	ExoDNA (internal)	Plasma	ExoDNA could be an interesting tool to diagnose pancreatic malignancies	Yang et al., 2017
*KRAS*	All stages	ExoDNA (internal) and ctDNA	Plasma	MAF > 5% is correlated with worse DFS and OS	Bernard et al., 2019

*MAF, mutant allele frequency. ExoDNA, exosomal DNA. Exo-DNA internal, a DNAse step was carried out prior to tumor DNA detection in exosomes. DFS, disease free survival. OS, overall survival. ctDNA, circulating tumor DNA. cfDNA, cell-free DNA. EV, extracellular vesicles. gDNA, genomic DNA. dsgDNA, double-stranded genomic DNA.*

Besides serum and plasma, other physiological or pathological body fluids contain EVs. A high clinical value was carried by pleural effusion EVs from lung cancer patients to determine the EGFR mutational status. Indeed, exosomal DNA *EGFR* mutational status correlated with gDNA from matched tumor tissues with 100% sensitivity, 96% specificity and 98% coincidence rate (Qu et al., 2019). More modestly, a comparative genomic profiling of solid bladder tumor DNA and matched urinary exoDNA from nine patients revealed a 65% (12/17) concordance rate for somatic mutation detection. ExoDNA and tumor samples presented a similar pattern of copy number aberrations (Lee et al., 2018).

Other molecular features of tumors can be identified by EV-DNA analysis. Frameshift mutation patterns in microsatellite stretches of *TGFBR2* and other microsatellite instability (MSI) target genes were found in the DNA cargo of MSI^+^ HCT-116 colorectal cancer cell line-derived exosomes Fricke et al., 2017. Thus, tumor MSI status could potentially be determined by EV-DNA analysis.

### Tumor-Specific Epigenetics

Yamamoto et al. (2016) analyzed the methylation status of BarH-like 2 homeobox protein (*BARHL2*), a gene known to be hypermethylated in gastric cancers (GC), in gastric wash-derived DNA and/or gastric juice-derived exoDNA of GC and healthy patients. Deeper analysis revealed that the *BARHL2* methylation status provided an area under the curve of 0.923 with 90% sensitivity and 100% specificity for the discrimination of GC patients from non-GC controls. Furthermore, the analysis of *LINE-1* and *SOX-2* methylation status was performed using DNA extracted from both GC cell lines and GC patients’ gastric juice microvesicles. Similar levels of methylation for *LINE-1* and *SOX-2* were obtained in EV-DNA compared to GC cell lines or patients’ tumor genomic DNA (Yamamoto, 2014). Therefore, as observed for the mutational status and sequence variations, epigenetic modifications such as methylations are shared between EV-DNA and the gDNA of their tumor of origin. More cancers need to be examined to confirm these encouraging results.

## EV-DNA as a Clinically Relevant Biomarker

In cancer, exosomes can oppose or potentiate the development of an aggressive tumor microenvironment, and thus impact tumor progression and metastatic and clinical outcome (Tai et al., 2018). EVs have also become choice materials for translational studies especially as liquid biopsy tools. In addition, the non-invasive and potentially repetitive nature of their analysis is applicable to both diagnosis and cancer follow-up. Routine tissue biopsy mutation status has been useful to step forward in targeted therapy, while ctDNA was proposed for the diagnosis and tumor monitoring. Although important technical advances have improved the sensitivity of ctDNA detection, and the fact that numerous studies have explored its diagnostic and prognostic values, the balance between translational research and application to routine lab use remains low.

Analysis of circulating EVs from patients with prostate cancer found tumor-related DNA, in particular *TP53* and *PTEN* mutations [Table 1, *n* = 4 (Lázaro-Ibáñez et al., 2014)], further confirmed in very few samples from metastatic castration-resistant cancers (*n* = 4; Vagner et al., 2018). The relevance of plasma EV-DNA remained underrated because efforts were produced for ctDNA detection. Therefore, only a few studies have compared ctDNA and EV-DNA diagnostic performances. Using ctDNA as a “gold standard” of DNA-based liquid biopsy of PDAC, stem studies by Alvarez’s group demonstrated that *KRAS* mutation detection in EV-DNA was superior to ctDNA for prognosis on large cohorts (Allenson et al., 2017; Bernard et al., 2019). The clinical performance of EV-DNA was somewhat disappointing since diagnostic accuracies ranked between 35 and 69% when compared to tissue biopsy (Bernard et al., 2019; Allenson et al., 2017, respectively). However, EV-DNA showed relevance in PDAC management since a correlation with non-recurrence survival was found in both studies, but limited to patients with metastatic disease (Allenson et al., 2017; Bernard et al., 2019). Moreover, although of good prognostic value, exoDNA based on mutant *KRAS* detection with highly sensitive detection techniques might not be suitable alone for general population screening as it yields high false-positive rates (Allenson et al., 2017; Bernard et al., 2019). More work needs to be carried out to associate this detection/quantification with another marker to increase specificity, such as combined liquid biopsy approaches (Cohen et al., 2017; Buscail et al., 2019a,b). Yang et al. (2017) highlighted the diagnostic value of PCR detection of *KRAS*^*G12D*^ and *TP53*^*R273H*^ that could differentiate healthy controls from patients with pancreatic cancer. This method, however, showed low performance to discriminating non-invasive (e.g., chronic pancreatitis, *n* = 9 and pancreatic cysts, *n* = 12) from malignant pancreatic pathologies. The very low number of patients makes definitive conclusions very risky, though. A high concordance rate (>95%) was found between circulating cfDNA (ct or EV) and primary tumors (Bernard et al., 2019) in a good size cohort.

According to the anatomical position of the primary tumor or its metastases, other biological fluids may be relevant for EV-DNA-based liquid biopsy. Interestingly, EVs carrying the *IDH1*^*G395A*^ mutation emitted by glioblastomas could cross the blood–brain barrier and be assayed intact in the bloodstream (García-Romero et al., 2017). Patients with mutated *IDH1* have a better prognosis than individuals with the wild-type allele, but with similar histology. In the same way, the genetic alterations of plasma or urine ctDNA and urine EV-DNA matched the mutational profiles of primary tumors in urothelial bladder carcinoma (Lee et al., 2018). The typical amplifications of *MDM2*, *ERBB2*, *CCND1*, and *CCNE1* and deletions of *CDKN2A*, *PTEN*, and *RB1* were characterized in only nine patients. More recently, exoDNA performed better for the diagnostic of non-small cell lung cancer (NSCLC) compared to EV-excluded cell-free DNA in bronchial wash fluid by the detection of *EGFR*^*T790M*^. The overall detection sensitivity of *EGFR* mutation was 89.7 and 31%, respectively, with 100% specificity (Park et al., 2020).

In conclusion, although very preliminary, combined recent analyses propose EV-DNA as an alternative to reach the promises of ctDNA, but more work is needed and more cancers must be evaluated to reach a final decision on whether to consider clinical lab development of this emerging biomarker.

## Conclusion and Future Developments

In the era of personalized medicine, molecular and epigenetic cancer profiling, using primary tumor tissue, takes up an ever greater place in a patient’s management and care. These modern molecular analyses help diagnosis, prediction of disease progression, and also adaptation and optimization of therapeutic decisions. Exosomal and EV-associated DNA seem promising as circulating biomarkers for cancer profiling as they can reflect the primary tumor and its metastases.

The mechanisms involved in active DNA release by tumor cells remain elusive; in particular, the way DNA species are sorted into EVs is far from being resolved. The recently reported amphisome-driven secretion in non-vesicular particles (Zhang et al., 2018) needs independent confirmation. In the same way, the final location of vesicle-associated DNA deserves more in depth exploration since EV-DNA can be bound to the outer lipid layer of EV membranes (Németh et al., 2017). Moreover, intra-EV tumor DNA might be less frequent than ctDNA (Klump et al., 2018). Therefore, the best extracellular compartment for tumor DNA detection and quantification is still debated.

The functional representation of EV-DNA of tumor behavior might also be partly diminished by the fact that a large part of ctDNA within cfDNA is released by dying tumor cells. This complexifies its cancer-relevant analysis. Furthermore, more work needs to be done to determine whether cfDNA is a good surrogate of tumor heterogeneity. Lee et al. (2018) reported the detection of additional somatic mutations in urinary exoDNA compared to bladder primary tumor DNA, suggesting that, although present in tumors, these mutations were missed by standard tissue biopsy genome analysis. However, those observations must be considered carefully because the authors increased the depth of exoDNA sequencing by 2.6 folds compared to tissue DNA analysis, which allows the detection of very rare (relevant?) mutations. In the same way, it will be necessary to determine which between the intraluminal and surface associated DNA better reflects tumor heterogeneity or tumor entire genome.

A major common limitation of studies interested in the clinical value of tumor EV-DNA is the minimal size of the patient’s cohorts (from as little as a few patients), reflecting that this topic is still emerging in the liquid biopsy field. By contrast, research on CTCs (circulating tumor cells) and ctDNA resulted in some indications for routine assessment. The other crucial development needed in the field is to implement a sufficient number of studies comparing the clinical values (with sufficient numbers of patients) of EV-DNA and ctDNA. Up to now, such kinds of studies are limited to PDAC and CRPC. Further external validation by other teams and in more cancers are needed.

One of the latest developments in tumor genetics is the characterization of epigenetic marks associated to tumor progression and/or severity (Costa-Pinheiro et al., 2015). Tumor epigenetic marks can as well constitute biomarkers with a theranostic value, since they permit the identification of targetable gene alterations and can be pharmacologically modified as personalized medicine approaches (see review Li et al., 2019). The extension of this knowledge to EV-DNA might be of particular interest to test whether EV-transferred DNA retains the epigenetic marks. If so, it would be worthwhile to ask whether they are stably kept during the journey and transferred in and impact recipient cells. Moreover, the traceability of EV can definitely link the primary tumor origin of epigenetic marks. Finally, finding the retention of tumor epigenetic marks in tumor EVs might position tumor EV-DNA as the ultimate circulating tumor biomarker, keeping both typical marks of tumor DNA: sequence variations and epigenetics.

Taken together, this review highlights the budding value of EV-DNA approaches to test and follow tumor liquid biopsies. Obviously, more work is needed with robust methodology to ascertain the clinical relevance of tumor EV-DNA as actual cancer biomarkers. However, the available literature tends to consider this approach very promising.

## Author Contributions

SA, SD, and EB involved in the manuscript writing, figure design, and table conception. VV, CD, JB, AB, J-PM, and FM-G performed the literature searching, articles selection, discussion on mechanisms and identification of DNA contents, manuscript editing, and final approval. LB, BB, and CL performed the manuscript organization, figure design, search of literature on clinical aspects, discussion on clinical relevance, manuscript editing, and final approval. All authors contributed to the article and approved the submitted version.

## Conflict of Interest

The authors declare that the research was conducted in the absence of any commercial or financial relationships that could be construed as a potential conflict of interest.
